# Network-Based, Cross-Sectional Analysis of Drug-Related Problems Reveals a Strong Association of Possible Inappropriate Medication and Clinical Outcomes in Romanian Elderly Nursing Home Residents

**DOI:** 10.3390/medsci14030359

**Published:** 2026-06-29

**Authors:** László-István Bába, Hanna Sebesi, Zsolt Gáll, Melinda Kolcsár, Soma Dávid, Noémi Eliza Medvés, George Jîtcă

**Affiliations:** 1 Department of Pharmacology and Clinical Pharmacy, Faculty of Pharmacy, George Emil Palade University of Medicine, Pharmacy, Science, and Technology of Targu Mures, 540142 Targu Mures, Romania; laszlo.baba@umfst.ro (L.-I.B.); melinda.kolcsar@umfst.ro (M.K.); george.jitca@umfst.ro (G.J.); 2 Department of Pharmacology, Faculty of English Medicine, George Emil Palade University of Medicine, Pharmacy, Science and Technology of Targu Mures, 540142 Targu Mures, Romania; hanna.sebesi@umfst.ro; 3 Faculty of Pharmacy, George Emil Palade University of Medicine, Pharmacy, Science, and Technology of Targu Mures, 540142 Targu Mures, Romania

**Keywords:** nursing home residents, drug–drug interactions, herb–drug interactions

## Abstract

Background/Objectives: Polypharmacy is common in elderly nursing home residents (NHR), due to the high prevalence of chronic diseases. This practice increases the risk of clinically significant drug–drug interactions (DDIs) with serious consequences for patient health and safety. The objective of this study was to evaluate the prevalence of DDIs using the UpToDate Drug–Drug Interaction Checker, potentially inappropriate medication (PIM, as defined by the STOPP-START criteria), and their association with major clinical outcomes. Methods: Demographic data, clinical history and detailed medication records of 275 patients from Romania were collected. Potentially inappropriate medications were identified using 16 selected criteria from the 2023 STOPP/START guidelines. Statistical analysis was performed using GraphPad, R, and Python, involving Chi-squared and Fisher’s exact tests with Benjamini–Hochberg correction, linear regression, and drug-interaction network analysis to characterise interaction frequency and severity. Results: Detailed medical histories over the past year were available for 76 patients. The mean number of drugs prescribed was 9.61 ± 4.47 drugs, with an average of 10.68 ± 10.54 potential interactions per patient. The primary clinical outcome was associated with not respecting certain STOPP-START recommendations (*p* < 0.01). Overall, 33.1% of NHRs utilised herbal supplements, resulting in a total of 76 potential herb–drug interactions. Conclusions: The results suggest a potential impact of DDIs on clinical outcomes, underscoring the need for further studies to clarify causality. The use of STOPP/START recommendations and deprescribing could lead to better tolerability and smaller drug-related burden in the institutionalised, elderly population.

## 1. Introduction

Medication-related problems in the elderly represent a major clinical concern due to the rapidly growing aging population. The number of people aged 65 and above is projected to more than double from the 761 million estimated in 2021 to more than 1.6 billion in 2050 [1]. The number of the oldest old is increasing even more rapidly, since the number of 80+ people is expected to triple by 2050, reaching an estimated 459 million worldwide.

Since the prevalence of multimorbidity (≥2 chronic conditions) and the number of drugs used by patients increases with age, it is important to understand and analyse the underlying patterns and to highlight the weaknesses that should be improved [2,3,4].

Numerous scientific articles have explored drug-related problems in older adults, noting that a high number of interactions exist in this population. Underscoring the severity of this issue, another study that measured drug–drug interactions (DDIs) with a different methodology found that more than 50% of nursing home residents (NHRs) presented at least one DDI [5].

A systematic review of reported DDIs found a prevalence of 29.7% (95% CI: 27.8–31.6%) NHRs [6]. Drug-induced problems in this elderly population are of great concern, since earlier observational studies reported a considerable increase in hospitalisation risk and mortality in certain sub-populations [7,8].

The critical need to address potentially inappropriate medications (PIMs) and DDIs is reinforced by clinicians’ readiness to resolve these issues following expert advice, which effectively averts potential adverse effects. In this regard, interventional studies on DDIs reported promising results regarding the impact on clinical outcomes. A study published by Lenander et al. found that 80% of general practitioners accepted the proposed improvements made by a clinical pharmacist in Sweden [9]. Similar results were found by the team led by Zermansky in the UK (75.6% acceptance and 76.6% of accepted recommendations were implemented) [10]. They also observed a significant reduction in the number of falls in the intervention group. No difference has been observed, however, in terms of drug costs, hospitalisation, mortality, and standardised mini-mental state examination scores [10].

Interventions on NHRs also show promising results [11,12]. A study that evaluated the impact of psychotropic drug regimen (anxiolytic, hypnotic and antipsychotic) revisions by pharmacists found that the number of potentially inappropriate psychoactive medications was considerably lower at 12 months after intervention [11]. Surprisingly, in the mentioned study, the authors did not find any significant difference in the number of falls between the two groups.

While DDIs have been more extensively studied, herb–drug interactions (HDIs) are gaining increasing attention due to their rising incidence. The widespread use of herbal supplementation was underscored by a nationwide survey conducted in Germany, which found that 60% of the public reported using herbal supplements. Even though these compounds may seem harmless to many, their misuse can lead to dangerous adverse effects [13].

NHRs are at an even higher risk of suffering from the underestimated effects of HDIs. Firstly, these patients commonly suffer from multiple chronic conditions, requiring complex pharmacotherapy, which alone increases the risk of DDIs. Secondly, patients with dementia frequently struggle with anxiety and sleep disturbances, both of which are often treated with herbal supplements. Lastly, these over-the-counter products often have very suggestive names that do not reflect their main components, which may lead patients to unknowingly take multiple doses of the same plant derivative. *Valeriana officinalis* and *Passiflora* spp. are among the most common ingredients found in both sleep-aiding and anxiety-relieving products [14].

A systematic review by Agbabiaka et al. reported that the prevalence of herbal product (HP) use varied greatly among the included reports (5.3–88.3%) and that the most frequently co-prescribed drugs with HP were antihypertensive drugs, β-blockers, diuretics, antihyperlipidemic agents, anticoagulants, analgesics, antihistamines, antidiabetics, antidepressants and statins [15]. The most important HDIs were caused by ginkgo biloba, garlic, ginseng and St. John’s wort (*Hypericum perforatum*), with the most important potential problem being an increased risk of bleeding [15]. In addition to these, *Valeriana officinalis* and *Passiflora* spp.-based products could also be of concern as these influence platelet function and inhibit certain CYP enzymes and P-glycoproteins [16]. This could potentially lead to clinically relevant adverse events. More than that, herbal products and supplements of other types might also be of concern in certain clinical situations [17].

The multitude of drugs used by a patient could be imagined as a complex system that could potentially interact with each other causing secondary problems (adverse drug reactions and interactions of different types). One could use mathematical models to study this system. Network science fits particularly well for this purpose. Within this framework, drugs and supplements could be imagined as nodes while edges could be DDIs and HDIs that connect these nodes. While traditionally applied to social networks, graph theory offers a powerful yet underutilised tool in clinical pharmacology [18]. With this novel, hypothesis-forming approach we aim to discover the critical drugs that could potentially cause numerous problems not only by the interaction burden itself, but also by the probability of connecting multiple interactions. We term these drugs bridge drugs.

The present work aimed to explore the clinical significance of potentially inappropriate medications (PIMs, defined as STOPP/START criteria [Screening Tool of Older Persons’ Potentially Inappropriate Prescriptions/Screening Tool to Alert Doctors to Right Treatment] problems, DDIs, or HDIs) in terms of the major clinical outcomes (falls, hospitalisations, fractures and surgical interventions) in NHRs in Romania.

## 2. Materials and Methods

### 2.1. Patients, Inclusion and Exclusion Criteria

The present study had a hybrid design. We used a retrospective analysis for outcome data (one year prior the inclusion) and cross-sectional study (for the drug and supplement use). This design enabled us to capture both the actual medication and the co-occurring adverse events one year prior to inclusion.

Demographic data, clinical history and medication records of 275 patients were collected by the authors from three nursing homes across Mures County, Romania. The nursing homes included in the study were various in terms of size (one small [<50 beds], one medium [50–100 beds] and one large [>100 beds]), all private-owned skilled nursing facilities (some of the patients had severe cognitive and/or physical impairments).

#### 2.1.1. Inclusion and Exclusion Criteria

**Figure 1:**
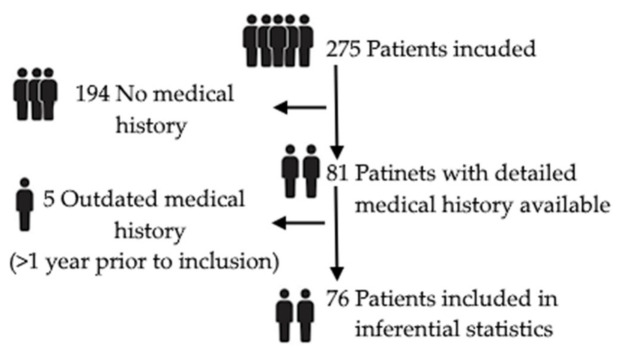
Exclusion flowchart of patients in study.

The inclusion criteria for the overall descriptive study were: older adults (no specific age restriction was applied, all NHRs were enrolled) residing in the participating facilities during the study period, for whom complete current medication administration records were available. No strict exclusion criteria were applied for the cross-sectional evaluation of drug-related problems. However, for the inferential analysis evaluating the association of medications with clinical outcomes (falls, fractures, and hospitalisations), a specific exclusion criterion was applied: the absence of a complete, 1-year retrospective medical and caregiver history. The exclusion flowchart is provided in Figure 1.

#### 2.1.2. Handling of Missing Outcome Data

Outcome data were missing for 199 patients (72.4%) entirely due to institutional data access limitations, as two out of the three study centres refused to provide full retrospective medical and hospitalisation histories for privacy reasons. Consequently, missing outcome data were handled using a complete case analysis approach, restricting the inferential outcome statistics exclusively to the 76 patients with fully available records. To assess the potential selection bias introduced by this missing data mechanism, a comparative analysis of demographic and clinical characteristics between the included (*n* = 76) and excluded (*n* = 199) subjects was conducted and is provided in Supplementary Table S1.

### 2.2. Primary and Secondary Exposure and Outcome Association

As the most important medication optimisations in the elderly population are the START/STOPP recommendations, we considered the violation of these the primary exposure. In addition to these, a number of PIMs were considered as secondary exposures in our analysis. These include X-type or D/X-type DDIs/HDIs according to the UpToDate classification, >4 ATC classes, the concomitant use of >8 drugs, presence of potential HDI and herbal supplement use. These exposures were all included in the primary inferential analysis for association. To identify other factors that could potentially be associated with worse outcomes, we included a set of secondary exposures (disease states and other alterations). The complete list of these is provided in Supplementary Figure S1.

The primary clinical outcomes recorded in this work over the one-year period prior to inclusion were falls, hospitalisations, fractures, and surgical interventions. For falls, standardised caregiver incident reports were used as the primary data source. Hospitalisations (acute hospitalisations, not considering regular or planned medical visits of all cause), fractures, and surgical interventions were extracted from the patients’ official medical records and discharge summaries.

A composite clinical outcome was also considered, which was defined as positive if any of the four aforementioned events occurred. The rationale for utilising this composite outcome was to provide a comprehensive measure of the burden of potential, drug-related problems, as well as to increase the statistical power for our exploratory analyses within this patient subset. Furthermore, for specific subsets there were insufficient data to undertake inferential analysis, justifying the use of a common category that describes all possible adverse outcomes.

### 2.3. Potentially Inappropriate Medication, Drug–Drug and Herb–Drug Interactions

Potentially inappropriate medication (PIM) was analysed using the STOPP-START criteria for elderly patients, according to the 2023 definition [19]. Although the STOPP/START criteria (version 3) encompass a comprehensive list of potentially inappropriate medications, applying all of these criteria was unfeasible due to the lack of access to specific laboratory parameters (e.g., precise eGFR, serum potassium) and detailed diagnostic timelines for all NHRs (especially for those that had been included in the inferential statistics). To mitigate misclassification bias, an internal expert panel of clinical pharmacists and a pharmacologist carefully reviewed the criteria. We selected a reduced set of 16 criteria (Table 1) based on two scientific principles: (1) unambiguous assessability using only the available medication administration records and documented diagnoses, and (2) high clinical relevance to our predefined primary outcomes (falls, fractures, hospitalisations and possibly surgical interventions). The main point for the latter was the ‘K’ criteria since these are specifically screened for drugs increasing fall risk, while the ‘C’ criteria focus on severe bleeding risks, since these are well-known causes of acute hospital admissions in this frail population [20,21,22]. As these criteria provide a comprehensive set of validated recommendations for improving the elderly patient drug regimen, the main focus is on deprescribing and avoiding certain drug classes. Thus, in our analysis, the STOPP criteria were used throughout.

Furthermore, DDIs and HDIs were identified using UpToDate’s Drug Interaction Checker and classified in four risk rating classes according to the following classification: A = No Known Interaction; B = No Action Needed; C = Monitor Therapy; D = Consider Therapy Modification; X = Avoid Combination [23].

While numerous HDI and DDI classification and grading tools are available, we used the UpToDate^®^ drug interaction checker for our analysis. This tool was developed by an international expert panel and comprehensively covers specific drugs used in Romania. As such, it classifies the various interactions from three perspectives for better understanding: risk rating, severity and reliability rating. Therefore, the use of this classification enabled us to evaluate the drug regimen in a standardised, scientifically rigorous yet reproducible manner.

### 2.4. Statistical Analysis

GraphPad (version 5, GraphPad Software Inc.; San Diego, CA, USA), R (version 4.4.2, R Foundation for Statistical Computing, Vienna, Austria), and Python (version 3.13.5) were used to perform statistical analysis. To find out the impact of the different risk factors on the outcomes, a series of Chi-squared and Fisher’s exact tests were run with family-wise correction (Benjamini–Hochberg correction for false discovery rate) for *p*-values. All other tests had the significance level set at α = 0.05. Additionally, linear regression analyses were undertaken to analyse the relationship between continuous independent predictors and Boolean outcomes using GraphPad Prism (GraphPad Software Inc.; San Diego, CA, USA). A network of drug–drug interactions was constructed using R with drugs as nodes and interaction severity and count as edge parameters. In this framework, node-level parameters (node degree and weighted degree centrality measures), describe individual drugs, while edge-level parameters are related to drug–drug interactions (interaction frequency and severity degree). Network-level parameters are related to the network of interactions as a whole (network density, connected components, and clustering coefficient). By calculating centrality measures—weighted degree, closeness centrality, betweenness and eigenvector—we could observe influential drugs within this network.

To address the high proportion of missing outcome data (72.4%, *n* = 199), a machine learning-based imputation sensitivity analysis was conducted on the full sample (*n* = 275). Outcome variables (falls, fractures, hospitalisations, and surgical interventions) were imputed using a Gradient Boosting Classifier, an ensemble tree-based method introduced by Friedman (2001) [24] and implemented via the scikit-learn library (v1.5.2, Python). This approach has been validated for imputation of binary outcomes with high missingness rates and has been shown to outperform complete-case analysis in terms of bias and precision under both missing-at-random and missing-not-at-random conditions [25]. The model was trained independently for each target variable on patients with known outcome data, using all available clinical and pharmacological parameters as predictors, with outcome-derived scores excluded (inclusion flag, composite outcome, and compound drug-related problem score). Model parameters were set as follows: 300 estimators, maximum tree depth of 3, learning rate of 0.05, subsample fraction of 0.80, and minimum samples per leaf of 5, with a fixed random seed (42) to ensure full reproducibility. For included patients (*n* = 76) with missing outcome data, structural recoding was applied prior to imputation: absence of documentation was treated as absence of the event, consistent with the complete caregiver and medical record availability confirmed for this subgroup. All inferential analyses (Fisher’s exact tests, odds ratios, and 95% confidence intervals) were repeated on the imputed dataset (*n* = 275) and compared with the primary complete-case analysis (*n* = 76) to assess the robustness of the reported associations, after similar *p*-value adjustment (Benjamini–Hochberg adjustment for false discovery rate). Concordance between the two analytical approaches was evaluated for each exposure–outcome pair.

### 2.5. Ethical Commission

The study received ethical commission approval from the University of Medicine, Pharmacy, Science and Technology of Targu Mureș, Romania (3283/01.07.2024). Institutional consent was obtained for data access.

## 3. Results

### 3.1. Patient Characteristics and Number of Drugs Used

A total of 275 patients were included in our study sample with an average age of 79.6 ± 9.95 years. Participants presented with an average of 2.95 ± 1.95 comorbidities, and were prescribed a mean of 9.61 ± 4.47 drugs. Drug–drug interaction analysis identified 10.68 ± 10.54 potential DDIs per patient. In total, 53% of our patients were taking over-the-counter (OTC) drugs, while 29% had some type of herbal compound in their pharmacotherapy. Table 2 summarises patient demographics, findings regarding the pharmacotherapies applied, and their number and potential interactions. Sex-based differences were analysed for each category, using Student *t*-test, Mann–Whitney U test, and Chi-squared test as appropriate for the data distribution and variable type.

The comparison between the included and excluded subjects from the sample revealed a significant difference between sex and, age since the excluded subjects were more likely females (OR = 1.866 95% CI [1.07–3.21]) and slightly older (medians with IQR: 78.5 [69–84] vs. 83 [75–88]) than patients with complete medical history. The morbidity index (average number of disease per NHR) was slightly higher in the included subjects (means ± SDs: 3.42 ± 0.23 vs. 2.89 ± 0.13). The medication burden was similar in the two cohorts (No. of drugs used [medians with IQR]: 8 (6–11) and 10 (6–12)); similarly, the number of DDIs per patient did not differ significantly (7.8 (3–13) 8 (3–15)) for the included and excluded patients. Detailed information about comparison between the two cohorts is displayed in Supplementary Table S1.

Due to the high prevalence of supplement consumption (53%), these agents were categorised for comparison based on their active component (non-herbal vs. herbal), then stratified by their intended health benefit as described in Section 3.3.

### 3.2. Prevalence of Potentially Inappropriate Medications Based on STOPP/START Criteria

The distinct pattern of STOPP/START criteria violations is detailed in Table 3. Briefly, we observed the highest prevalence of A3 criterion violation (duplicate drug class prescription) both in males and females (14.29% and 11.41%, respectively). Numerous recommendations were fully respected (B18, C6, C10, C13, and K6).

### 3.3. Supplement Use in the Sample

Each supplement type utilised by 4 or more patients was categorised individually; the remainder were classified as ‘other’. Of the 11 specific categories, the most prevalent were multivitamins and minerals (25.45%), followed by fish oil (10.54%) and electrolyte supplements (10.18%). Statistically significant associations were observed between sex and the administration of calcium/vitamin D (*p* = 0.02), hepatoprotective supplements (*p* = 0.03), and agents categorised as other (*p* = 0.03).

Herbal supplements were taken by 29% of our sample; the most frequently used were ginkgo biloba (16.36%), silimarine (5.09%), and herb-based laxatives (4.72%). A statistically significant association was found between sex and the consumption of silimarine (*p* = 0.01). Within the specific Ginkgo-using sub-cohort (*n* = 45), a significant positive association was observed between the number of concurrent medications and the total number of HDIs. Linear regression analysis indicated that the number of co-administered drugs accounted for 28% of the variance in HDI occurrence (R^2^ = 0.28; *p* = 0.0087).

Analysis of the most frequently used herbs demonstrated a significantly higher number of potential HDIs in the valerian-treated group compared to the ginkgo-treated group (*p* = 0.02). The findings on supplement use are summarised in Table 4.

### 3.4. Exposures Associated with Major Adverse Events

**Figure 2:**
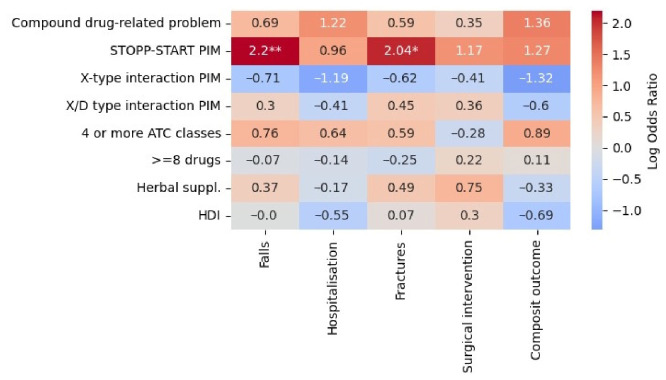
Heatmap representation of the association of different risk factors and the major clinical outcomes (*n* = 76). Red—positive association (log OR > 0–OR > 1); blue—negative association (log OR < 0–OR < 1). Benjamini–Hochberg correction for false discovery rate has been applied: adjusted * *p* < 0.05, ** *p* < 0.01. Significant results are summarised in Supplementary Table S3.

**Figure 3:**
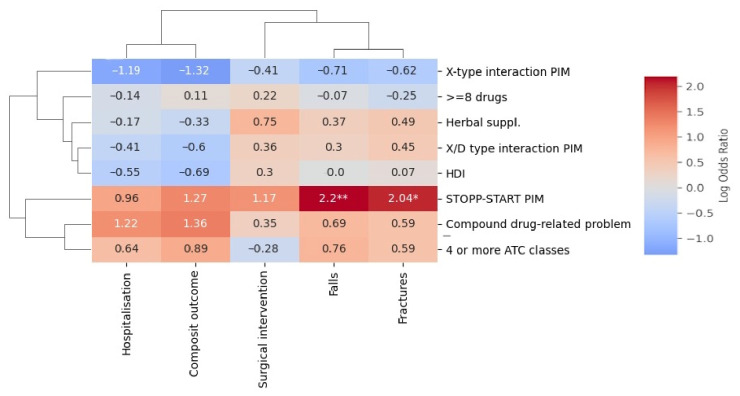
Hierarchical clustering of the association of different major risk factors and the major clinical outcomes (*n* = 76). Agglomerative hierarchical clustering was used with the Euclidean distance metric and average linkage (UPGMA). Red—positive association (log OR > 0 that is OR > 1); blue—negative association (log OR < 0 that is OR < 1). Benjamini–Hochberg correction for false discovery rate has been applied: adjusted * *p* < 0.05; ** *p* < 0.01. This tree structure groups variables based on their co-occurrence. Shorter linkage distances indicate variables that frequently appear together in the patients (e.g., fractures and fractures).

The analysis of association of different risk factors with the clinical outcomes revealed several factors that had distinct roles. Significant association was found between primary exposure (breaches in the STOPP/START recommendations) and falls and fractures, respectively. Both of these events show a strong, positive association (OR [95% CI] 9.02 [2.45–32.35] and 7.67 [2.03–27.81]), respectively (Figure 2 and Figure 3). To ensure transparency given the limited cases in the association analysis, we provide the absolute numbers of patients stratified by the four clinical outcomes in Supplementary Table S2.

No other pre-specified exposure reached statistical significance after Benjamini–Hochberg correction. The complete results of all exposure–outcome association tests, including odds ratios and adjusted *p*-values for all variables examined, are provided in Supplementary Figure S1.

Other factors showing notable, though non-significant, associations with several outcomes included vascular dementia, which was associated with increased odds of hospitalisation and the composite outcome, as well as epilepsy and other convulsions, which were associated with increased odds of fractures and surgical interventions. Electrolyte disturbances and blood cell count problems during the last hospital visit approached statistical significance after correction for false discovery rate, increasing the odds for hospitalisation and the composite clinical outcome (OR = 4.73; 3.05 and 3.26, respectively, adjusted *p* between 0.06 and 0.09, Supplementary Figure S1).

To interpret the similarities, identify possible collinearity among outcomes, and assess differences between outcome clusters, we undertook a hierarchical clustering of outcomes and risk factors using seaborn in Python. The results of the clustering are shown in Figure 3.

### 3.5. Imputed Data Analysis

To formally assess the robustness of the primary inferential analysis (complete cases, *n* = 76) and address the potential for selection bias introduced by the 72.4% missing outcome rate, a gradient boosting-based imputation sensitivity analysis was performed on the full sample (*n* = 275), as described in Section 2.4.

The analysis of this set of data confirmed the existence of a positive, significant association of STOPP-START breaches and falls (OR = 3.958 [1.61–9.7], *p* < 0.024), while the association between STOPP-START violations and fractures (OR = 2.21 [0.931–5.22], *p* = 0.31) did not reach statistical significance.

A thorough analysis of the imputed cases revealed several associations that were not significant in the complete-case analysis. Therefore, we consider these only hypothesis-forming risk factors that should be addressed by subsequent real-world observations. These include the following: STOPP-START violations with surgical interventions and hospitalisations; concurrent use of >8 drugs/supplements on a regular basis with falls. Furthermore, several diseases and laboratory findings were found to be associated with adverse clinical outcomes (type II diabetes mellitus, haematological abnormalities, electrolyte or other laboratory disturbances). Supplementary Table S4 displays selected pairs of exposures and adverse outcomes with possible clinical significance.

### 3.6. Graph-Based Analysis of Drug Interaction Patterns

**Figure 4:**
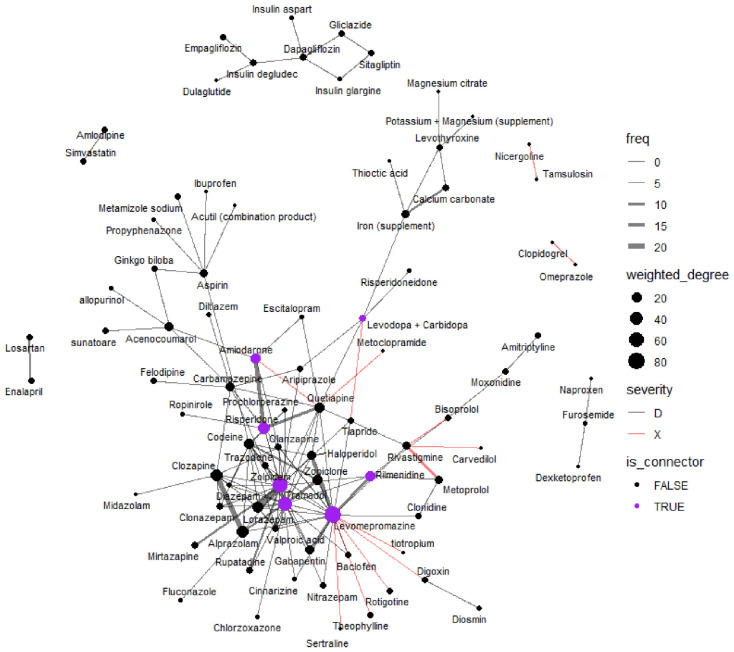
The graph representation of the interactions with severity grade D or X. Purple nodes represent bridge nodes between different clusters. The network is obtained from the whole sample (*n* = 275). Node size reflects the weighted degree (number of interactions a certain drug has in the whole sample). Purple nodes represent bridge nodes, which act as critical connectors between otherwise isolated pharmacological clusters.

**Figure 5:**
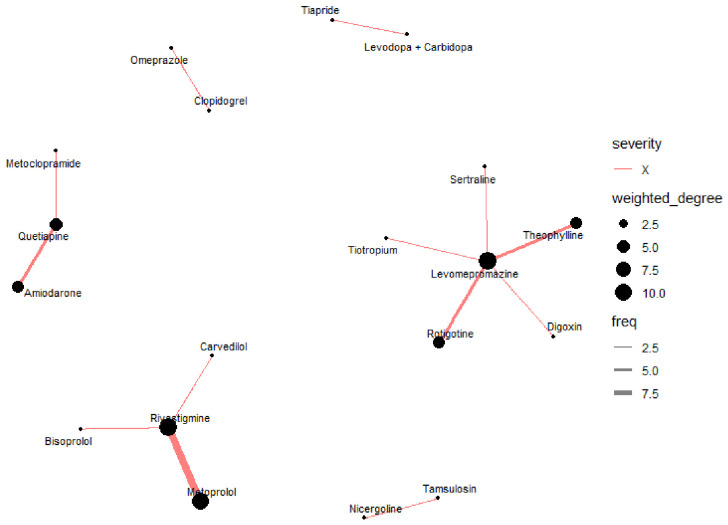
Graphs showing clustered X-type interactions in the sample (*n* = 275). The most important nodes that connect these are levomepromazine, quetiapine and rivastigmine. Red edges represent serious DDIs (X-type interactions). Edge weight (number of interaction count in cohort) is represented by thickness. Node size represents weighted degree.

In an effort to identify drugs that form clusters with greater risk for adverse events, we undertook a graph-based analysis of the identified interactions using R (tidygraph and ggraph packages) (Figure 4 and Figure 5). From a clinical perspective, the more severe interactions (X and D severity grades) impose a considerable burden. More than that, the complexity of an exhaustive graph containing all interactions is often populated with a large number of low-severity-grade interactions. Thus, we undertook two different sets of analyses on the network of interactions. First, we undertook an analysis of the net of interactions having severity grades D or X. After that, we repeated the same set of analyses on all interactions (severity grades A-X). The result of the first analysis is reported in Table 5, while the results of the second analysis are displayed in Supplementary Table S5.

Within this framework, we defined clusters using the Louvain method with connection numbers (weighted degree) as weights. This method partitions a network into clusters by maximising modularity (i.e., the excess of within-cluster connections relative to what is expected under random connectivity). Node degree represents the number of distinct drugs that a drug is connected to, while weighted degree represents the sum of all interactions a drug has. We also quantified the closeness centrality (how readily a drug can form clusters by reaching other drugs through DDIs—the higher the number, the more the risk increases), betweenness centrality (how often a drug connects separated clusters), eigenvector centrality (the measure that quantifies how many important drugs a certain drug is connected to), and the number of clusters connected (how many distinct clusters appear in a drug’s neighbourhood). Bridge drugs were defined as drugs with a betweenness centrality at or above the 90th percentile and whose local neighbourhood connected at least two distinct network clusters. These results are displayed in Table 5.

Analysis of D and X severity interactions revealed a large cluster of interactions containing mainly central nervous system-acting drugs. Within this cluster, an important node is levomepromazine. As represented in Figure 5, this drug is also a bridge drug in the network of X-type interactions, meaning that this drug connects several clusters, resulting in a whole sub-net of interconnected drugs. Tramadol acted similarly as an important hub, showing a broad network of connections across our sample. Zolpidem, tramadol, risperidone, alprazolam, clozapine and lorazepam also presented a high number of interactions (mainly of category D, shown in Figure 4), while category X interactions (red) were the most prevalent among rivastigmine, metoprolol and quetiapine (Figure 4 and Figure 5). Seven bridge drugs were identified among X- and D-type interactions (Figure 4 and Table 4). These were the following: levomepromazine, zolpidem, tramadol, risperidone, amiodarone, rilmenidine, levodopa + carbidopa. We observed great differences in the way prescribers used these drugs, and the prevalence of their use was relatively low in cases where outcome data were available, and we were not able to undertake meaningful inferential statistics for these drugs in terms of the impact on the major outcomes included in our study.

## 4. Discussion

In the present work, we aimed to conduct an exploratory assessment of PIMs (predicted DDIs, HDIs and STOPP/START criteria breaches) and to evaluate their observational association with four major clinical outcomes (falls, fractures, hospitalisations, and surgical interventions within one year prior to inclusion). We found that NHRs in Romania use an average of 9.61 ± 4.47 drugs on a regular basis. This exceeds the standard definition of polypharmacy (>5 drugs) and approaches levels called hyperpolypharmacy or excessive polypharmacy (>10 drugs), which was observed in a substantial portion of our 275-patient cohort (15.6%, *n* = 43). These findings are closely aligned with a large-scale study by Josendal et al., which examined elderly patients in Norway and found an even higher average of 10.6 drugs/patient [26]. Other articles have reported similarly high drug numbers in institutionalised elderly patients. A systematic review from 2023 reported an average number of drugs between 3 and 10.1; most studies included in this systematic analysis reported 8 or more chronically used drugs per patient [27]. Similarly, a post hoc analysis of the INCUR study reported an average drug number of 8.5 ± 4.1 in a French sample of 800 nursing home residents [28]. Though not consequently defined, polypharmacy is commonly defined as the concomitant use of five or more drugs. Reported data in the literature varies greatly in terms of prevalence and definition (4–96.5%) in different settings (depending on the age group, definition used, etc.) [29].

Despite of numerous reports from other regions, data from Eastern European nursing home populations, particularly Romania, remain scarce, and it is unclear whether previously reported associations between PIMs and clinical outcomes generalise to this setting. Nevertheless, our results fit to the observed trend regarding the reported drug usage in the elderly, with 87% of patients using ≥5 drugs and 49% using ≥10 drugs on a regular basis (Table 2).

Our findings show that 29% of NHRs utilised herbal supplements, with 76 potential HDIs. While this number is substantially lower compared to herbal supplement usage in Germany (60%), the selection of herbs in our cohort, like ginkgo, valerian and passionflower, is highly reactive due to their complex pharmacology [13]. Specifically, 9.2% (*n* = 28) of all identified interactions were predicted to be directly related to the coadministration of these herbs with synthetic drugs. Notably, when comparing the interaction profiles of the most commonly utilised herbs, a Mann–Whitney U test revealed a significantly higher number of possible HDIs per patient in the valerian-treated group compared to the ginkgo-treated group (means ± SD: 1.5 ± 0.97; 0.65 ± 0.83, respectively; U = 60.5; *p* = 0.02). Furthermore, ginkgo usage accounted for 28% of the variance in HDI occurrence (R^2^ = 0.28; *p* = 0.0087), indicating its potential to cause predicted HDIs. Since our preliminary results came from an unadjusted regression model because of the size of the cohort, caution is advised to avoid speculation. Nevertheless, the role of this increase in the number of interactions is not clear and pleads for further analysis.

Regarding the similarities of different outcomes, we identified two distinct clusters. First, fractures and surgical interventions clustered tightly; epilepsy and other convulsions showed a strong positive association with these injuries, appearing as closely related risk factors in the dendrogram (Figure 5). This underscores the high burden of the causative disease and possibly the medication that is used to control convulsions. Indeed, the use of anti-epileptics is known to cause an increase in the incidence of falls, fractures and other adverse clinical outcomes [30]. It is worth underscoring that the extent to which these two contributors (i.e., the disease itself and the drugs that are used to control them) add to the final association is unknown; therefore, caution is advised on interpretation of these results.

Second, major similarity was found between vascular dementia and type II diabetes, dyslipidaemia, Alzheimer dementia’s association with hospitalisation and composite clinical outcome. Furthermore, this trend extended to adverse clinical effects, where PIMs showed similar patterns in terms of falls and fractures.

Deviation from the STOPP/START recommendations were shown to be significantly associated with a higher history of physical trauma, specifically falls and fractures, within the study population. However, given the retrospective nature of the outcome data, this association must be interpreted with caution. We cannot rule out the possibility of reverse causation—that is, the occurrence of these adverse clinical events may have subsequently influenced prescribing patterns. Therefore, our findings should be viewed as hypothesis-generating rather than establishing a direct causal impact. On the other hand, these results are consistent with previous observations reported by Hill-Taylor et al., further validating the use of these criteria as essential safety tools [31]. The identification and resolution of drug-related problems is also emphasised by numerous data from the literature. A narrative review from 2010 reported that there is solid evidence in the literature (15/18 articles) regarding the effectiveness of the various interventions undertaken, leading to improvement in at least one aspect of suboptimal prescribing [12]. Newer reports and systematic reviews similarly confirmed the efficacy of targeted deprescribing interventions and treatment optimisation in frail older adults and nursing home populations [32,33].

Although our limited sample size with complete follow-up data precluded inferential analysis for the individual STOPP/START criteria, our work offers insight into the prevalence of these in our frail population. The most frequent violation observed was the A3 criterion (12.36%), representing the unjustified duplication of drug classes. This practice potentially increases the drug-related burden without considerable therapeutic benefit. Furthermore, nearly 10% of our patients were subjected to ‘K’ category violations, which specifically flag the inappropriate use of fall-risk-increasing drugs, such as benzodiazepines, antipsychotics, and antidepressants [21]. The continuous prescription of these central nervous system-active agents in frail older adults is known to impair sensorium and balance, which provides a direct pathophysiological explanation for the strong association we observed between overall STOPP/START breaches and the high incidence of falls and fractures. Additionally, the observed ‘C’ category violations (3.64%) point to severe bleeding risks associated with inappropriate antithrombotic use, which is a well-documented driver of acute hospitalisations. Another observation that our work brings is the general prevalence of STOPP/START violations that was nearly 30% in our sample. This relatively high proportion and the observed association with physical trauma (falls and fractures) plead for the importance of this problem in an already frail population.

One of the most striking findings of the present work is the fact that in our sample, an average of 10.6 potential DDIs per patient were predicted. In the context of our exploratory network analysis, we identified multiple drugs (levomepromazine, quetiapine, and rivastigmine) acting as nodes, connecting several X-type DDIs. These nodes lie on the shortest path between several other therapeutic agents, meaning that their presence in the prescription could trigger a cascade of interactions across the entire medication regimen, eventually resulting in a high burden.

The complex network analysis visualised in Figure 2 and Figure 3 underscores the high interaction prevalence found in our sample size and urges us to think of drug–drug interactions as a web instead of isolated pairs. Our most prominent central hubs, levomepromazine, zolpidem, tramadol, risperidone, amiodarone, rilmenidine and levodopa + carbidopa, could possibly link multiple potentially dangerous interaction pathways, which suggests that their prescription in an already polymedicated geriatric patient should warrant additional caution. These might include dosage adjustments or careful monitoring or even switching to alternative options.

A recent graph-based exploratory analysis published by Radu et al. based on a Romanian cohort of hospitalised cardiovascular patients similarly analysed the network of DDIs to identify important drugs that act as central nodes within the network [34]. They found that furosemide, pantoprazole, spironolactone, bisoprolol, amiodarone, and perindopril occupied important positions within the network. In our exploratory analysis of X and D types of interactions, amiodarone also appeared as an important drug. More important drugs in our analysis were psychotropics, which occupied the first eight positions in descending order by weighted degree (Table 5). Our whole-network analysis revealed a similar pattern; notably, furosemide, indapamide, aspirin (acetylsalicylic acid), metoprolol, and bisoprolol were among the first ten most important drugs by weighted degree (Supplementary Table S4), which is highly consistent with Radu et al.’s top interacting agents. The differences in our top rankings might be caused by methodological disparities between the two studies. First, they analysed the medication of cardiovascular patients, while our patients were nursing home residents with a high prevalence of neuro-psychiatric disorders. Second, they used drugs.com as their DDI interaction checker. Nevertheless, our findings in the whole-network analysis are in strong accordance with their exploratory analysis.

Whether the presence of these bridge drugs actually increases the risk of adverse clinical outcomes could not be determined in this study due to insufficient sample size, and this should be addressed in future prospective research. From a practical clinical perspective, identifying these drugs in an elderly patient’s therapeutic scheme does not automatically mandate their deprescription. Rather, it offers a theoretical prioritisation tool for pharmacists and clinicians during comprehensive medication reviews. Prioritising the critical evaluation of these ‘bridge drugs’ could efficiently resolve multiple interaction pathways simultaneously.

Our study has several limitations. First, our study is limited by the fact that outcome data could only be retrieved for 76 of the 275 patients, creating a high risk of selection bias. Therefore, the inferential statistics presented here may lack broader generalisability and should be viewed primarily as a hypothesis-generating, exploratory analysis rather than confirmatory evidence. Additionally, the excluded patients were slightly older and were more likely females than males. Furthermore, a comparison of clinical characteristics revealed that the included cohort presented with a higher average morbidity (3.42 ± 0.23 vs. 2.89 ± 0.13) and was significantly younger (78.5 [69–84] vs. 83 [75–88] years) compared to the complete-case group (Section 3.1 and Supplementary Table S1). Because the excluded population was generally older and potentially more frail because of their higher morbidity, our complete-case analysis (*n* = 76) likely represents a slightly healthier or less complex sub-population. Regarding the direction and impact of this selection bias, it is highly likely that our current complete-case inferential statistics actually underestimate the true incidence of adverse clinical outcomes and the strength of their association with potentially inappropriate medications. Importantly, our gradient boosting-based imputation sensitivity analysis on the full sample (*n* = 275) confirmed the primary association, demonstrating that despite this conservative approach, our core exploratory findings remain robust.

Second, the number of patients that used the bridge drugs was too low to evaluate the association with the clinical outcomes and therefore further studies are urged to elucidate the potential role of these drugs in increasing the clinical burden by connecting DDI clusters. Again, this aspect underscores the fact that the present work has an exploratory and hypothesis-generating rather than confirmatory nature. Another important aspect is that the recorded outcomes were parallel in time with the exposure; hence, the reverse causation cannot be ruled out (i.e., new drugs are given because of an adverse event such as a recent hospitalisation). Nevertheless, our exploratory analysis suggests a potential link between exposure to PIMs and clinical outcomes, which warrants further analysis to assess causality.

Furthermore, the DDI identification tool used inherently brings weaknesses related to the quality of evidence, since the available interaction checkers utilise multiple layers of evidence in synthesis. In vitro studies, theoretical pharmacokinetic mechanisms, and isolated case reports are all considered. Consequently, the predicted HDIs vary in reliability rating. We emphasise that the identified DDIs and HDIs represent theoretical risks based on pharmacological databases, which do not necessarily translate to actual clinical adverse events in every patient.

The implications of the present study should be viewed within the aforementioned limitations. First, considering the high prevalence of supplement use and the numerous possible HDIs identified, we underscore the necessity of routine revision of OTC and supplement use, in particular those that contain ginkgo biloba or valerian. These were considerable drivers of predicted HDIs and the deprescription of these could potentially lead to lower drug-related burden. Our network analysis revealed that some drugs lie between several other drugs in the network; thus, their use potentially leads to a cluster of DDIs. We termed these ‘*bridge drugs*’ (in our sample, these include tramadol and levomepromazine). The continuous use of these drugs creates a theoretical vulnerability, as they could connect otherwise isolated DDI clusters. While our current sample size precluded the statistical linking of these specific bridge drugs to major clinical outcomes, this exploratory network approach sets the stage for future longitudinal experiments. Such studies could assess whether the targeted deprescription of these bridge drugs results in a reduction in adverse outcomes in the elderly population. Existing data in the literature confirm that the aforementioned bridge drugs are associated with a considerable clinical burden. For instance, increased mortality has been reported in association with levomepromazine, amiodarone, and tramadol, which emerged as important bridge drugs in our analysis [34,35,36,37,38]. However, subsequent prospective studies are required to establish a direct causal link between these drugs’ central structural role in the interaction network and the actual occurrence of adverse clinical events.

At the policy level, our results invite the implementation of a standardised, multidisciplinary review protocol based on the STOPP/START criteria upon nursing home admission. Implemented at a national level, this could lead to a significant reduction in drug-related problems.

## 5. Conclusions

Our study highlighted the importance of PIMs in terms of DDIs, STOPP/START breaches and HDIs. Notably, the concurrent use of herbal supplements, particularly valerian and ginkgo biloba, proved to be a significant driver of interaction risks within this vulnerable cohort. The use of STOPP/START recommendations and the deprescribing of *bridge drugs* could lead to better tolerability. This in turn could potentially mitigate the drug-related burden in the institutionalised, elderly population.

Furthermore, our network-based analysis successfully identified highly connected *bridge drugs* that structurally link multiple interaction clusters. Although the direct clinical impact of these specific medications remains unverified and requires robust testing in future longitudinal studies, identifying such central nodes offers a novel, theoretical framework in reviewing the complex therapeutic schemes in frail elderly patients.

Consequently, routine medication reviews must rigorously evaluate both synthetic polypharmacy and herbal supplementation to optimise patient safety.

## Figures and Tables

**Table 1:** The STOPP-START criteria included in the study (according to the 2023 definition—third edition) [19].

STOPP-START Criteria	Recommendation
A3	Any duplicate drug class prescription for daily regular use (as distinct from PRN use), e.g., two concurrent NSAIDs, SSRIs, loop diuretics, ACE inhibitors, anticoagulants, antipsychotics, opioid analgesics (optimisation of monotherapy within a single drug class should be observed prior to considering a new agent).
B3	Beta-blocker in combination with verapamil or diltiazem.
B12	Aldosterone antagonists (e.g., spironolactone, eplerenone) with concurrent potassium-conserving drugs (e.g., ACEI’s, ARB’s, amiloride, triamterene) without monitoring of serum potassium (risk of dangerous hyperkalaemia, i.e., >6.0 mmol/L–serum K should be monitored regularly, i.e., at least every 6 months).
B18	NSAIDs or systemic corticosteroids with heart failure requiring loop diuretic therapy (risk of exacerbation of heart failure).
C6	Ticlopidine in any circumstances (clopidogrel and prasugrel have similar efficacy, stronger evidence and fewer side-effects).
C10	NSAID and vitamin K antagonist, direct thrombin inhibitor or factor Xa inhibitors in combination (risk of gastrointestinal bleeding).
C13	Direct thrombin inhibitor (e.g., dabigatran) and diltiazem or verapamil (increased risk of bleeding).
C14	Apixaban, dabigatran, edoxaban, rivaroxaban and P-glycoprotein (P-gp) drug efflux pump inhibitors, e.g., amiodarone, azithromycin, carvedilol, cyclosporin, dronedarone, itraconazole, ketoconazole (systemic), macrolides, quinine, ranolazine, tamoxifen, ticagrelor, verapamil (increased risk of bleeding).
C16	Aspirin for primary prevention of cardiovascular disease.
D14	Drugs with potent anticholinergics/antimuscarinic effects in patients with delirium or dementia (risk of exacerbation of cognitive impairment).
D24	First-generation antihistamines as first-line treatment for allergy or pruritus (safer, less toxic antihistamines with fewer side effects now widely available).
I1	Systemic antimuscarinic drugs in patients with dementia or chronic cognitive impairment (risk of increased confusion, agitation).
K1	Benzodiazepines in patients with recurrent falls (sedative, may cause reduced sensorium, impair balance).
K5	Anti-epileptic drugs in patients with recurrent falls (may impair sensorium, may adversely affect cerebellar function).
K6	First-generation antihistamines in patients with recurrent falls (may impair sensorium).
K8	Antidepressants in patients with recurrent falls (may impair sensorium).

**Table 2:** Demographic characteristics of the subjects and the number of drugs and predicted drug–drug and herb–drug interactions (*n* [%], mean ± SD).

Characteristic	Male (*n* = 91)	Female (*n* = 184)	Overall (*n* = 275)	*p* Value *
Age	75.45 ± 12.26	78.14 ± 7.87	79.67 ± 9.95	U = 6070, *p* = 0.0002
Morbidity (number of diseases)	2.95 ± 1.95	3.13 ± 1.08	3.07 ± 1.85	U = 7742, *p* = 0.30
No. of drugs	9.57 ± 4.82	9.63 ± 4.29	9.61 ± 4.47	U = 8232, *p* = 0.82
No. ATC classes prescribed	3.33 ± 1.57	3.31 ± 1.30	3.32 ± 1.39	U = 8360, *p* = 0.98
No. of patients with ≥5 drugs/supplements	76 (84)	163 (89)	224 (87)	χ2 = 1.38 *p* = 0.24
No. of patients with ≥10 drugs/supplements	43 (47)	93 (51)	136 (49)	χ2 = 0.26 *p* = 0.61
No. of predicted DDIs per patient	11.73 ± 11.83	10.17 ± 9.81	10.68 ± 10.54	U = 8110, *p* = 0.67
No. of patients presenting herb–drug interactions	12	31	43	χ2 = 0.62 *p* = 0.43
No. of subjects taking supplements	49 (54)	98 (53)	147 (53)	χ2 = 0.01 *p* = 0.93
No. of subjects taking herbal supplements	28 (31)	51 (28)	79 (29)	χ2 = 2.42 *p* = 0.12
No. of patients with violations of STOPP/START criteria	26 (28.57)	56 (30.43)	82 (29.82)	χ2 = 0.16 *p* = 0.68
Average No. of STOPP/START criteria violations ± SD	0.07 ± 0.31	0.13 ± 0.44	0.11 ± 0.40	U = 8146, *p* = 0.42

* Student’s *t* and Mann–Whitney U test, and Chi-squared test comparing male and female subjects.

**Table 3:** STOPP/START violations in the whole sample (*n* = 275). Characteristics of the subjects and the number of drugs and predicted drug–drug and herb–drug interactions (*n* [%]).

	Male (*n* = 91)	Female (*n* = 184)	Overall (*n* = 275)	Association Analysis Significance (*p*) ^1^
A3	13 (14.29)	21 (11.41)	34 (12.36)	0.06
All B type	7 (7.69)	10 (5.43)	17 (6.18)	0.60
B3	0 (0)	1 (0.54)	1 (0.36)	>0.99
B12	7 (7.69)	9 (4.89)	16 (5.82)	0.41
B18	0 (0)	0 (0)	0 (0)	n.a.
All C type	1 (1.1)	9 (4.89)	10 (3.64)	0.17
C6	0 (0)	0 (0)	0 (0)	n.a.
C10	0 (0)	0 (0)	0 (0)	n.a.
C13	0 (0)	0 (0)	0 (0)	n.a.
C14	1 (1.1)	9 (4.89)	10 (3.64)	0.17
C16	0 (0)	0 (0)	0 (0)	n.a.
All D type	4 (4.4)	9 (4.89)	13 (4.73)	>0.99
D14	3 (3.3)	8 (4.35)	11 (4)	>0.99
D24	1 (1.1)	1 (0.54)	2 (0.73)	0.55
I1	0 (0)	1 (0.54)	1 (0.36)	>0.99
All K type	6 (6.59)	21 (11.41)	27 (9.82)	0.28
K1	4 (4.4)	13 (7.07)	17 (6.18)	0.44
K5	2 (2.2)	5 (2.72)	7 (2.55)	>0.99
K6	0 (0)	0 (0)	0 (0)	n.a.
K8	0 (0)	3 (1.63)	3 (1.09)	0.55
No violation	65 (71.43)	128 (69.57)	193 (70.18)	0.78
At least one violation	26 (28.57)	56 (30.43)	82 (29.82)	0.78
1 criterium	20 (21.98)	39 (21.2)	59 (21.45)	0.88
2 criteria	5 (5.49)	13 (7.07)	18 (6.55)	0.80
3 criteria	1 (1.1)	2 (1.09)	3 (1.09)	>0.99
4 criteria	0 (0)	2 (1.09)	2 (0.73)	>0.99

^1^—Fisher’s exact test *p* values for association of sex with occurrence of STOPP/START violations.

**Table 4:** Supplement use stratified by supplement type.

Supplement Type	Male (*n* = 91)	Female (*n* = 184)	Overall (*n* = 275)	*p* Value ^1^
Non-herbal supplements				
Electrolyte supplements	9 (9.89%)	19 (10.32%)	28 (10.18%)	χ2 = 0.01 *p* = 0.91
Probiotics	1 (1.09%)	5 (2.71%)	6 (2.18%)	*p* = 0.66
Calcium and/or Vit D	3 (3.29%)	22 (11.95%)	25 (9.09%)	*p* = 0.02
Multivitamins and minerals	22 (24.17%)	48 (26.08%)	70 (25.45%)	χ2 = 0.11 *p* = 0.73
Liver support	10 (10.98%)	8 (4.34%)	18 (6.54%)	χ2 = 4.39 *p* = 0.03
Phospholipids	1 (1.09%)	4 (2.17%)	5 (1.81%)	*p* = 1.00
Fish oil (omega 3)	6 (6.59%)	23 (12.5%)	29 (10.54%)	χ2 = 2.25 *p* = 0.13
Melatonin	4 (4.39%)	10 (5.43%)	14 (5.09%)	*p* = 1.00
Digestive enzymes	3 (3.29%)	3 (1.63%)	6 (2.18%)	*p* = 0.40
Eye health supp.	1 (1.09%)	3 (1.63%)	4 (1.45%)	*p* = 1.00
Urinary tract supp.	2 (2.19%)	3 (1.63%)	5 (1.81%)	*p* = 0.66
Other ^2^	8 (8.79%)	34 (18.47%)	42 (15.27%)	χ2 = 4.41 *p* = 0.03
Herb-based supplements				
*Ginkgo biloba*	17 (18.68%)	28 (15.21%)	45 (16.36%)	χ2 = 0.53 *p* = 0.46
*Passiflora*	3 (3.29%)	8 (4.34%)	11 (4%)	*p* = 1.00
Valerian	1 (1.09%)	8 (4.34%)	9 (3.27%)	*p* = 0.27
Herb-based laxatives	1 (1.09%)	12 (6.52%)	13 (4.72%)	*p* = 0.06
Silimarine	9 (9.89%)	5 (2.71%)	14 (5.09%)	χ2 = 6.48 *p* = 0.01

^1^ Chi-squared test or Fisher’s exact test for the association of sex and administration of different supplements. Where the incidence number was below five, Fisher’s exact test was performed (no test statistics reported). ^2^ The supplements that did not fit into any of the earlier categories and did not reach an observation count of at least four were considered collectively as the other type.

**Table 5:** The network-based analysis of the most important drugs (fifteen drugs with the highest number of interactions—weighted degree). Analysis of the whole sample (*n* = 275).

Drug	Degree ^1^	Weighted Degree ^2^	Closeness Centrality ^3^	Betweenness ^3^	Eigenvector ^4^	Number of Clusters Connected ^5^	Bridge Drug ^6^
Levomepromazine	24	82	1.01	1141.50	0.93	6	Yes
Zolpidem	21	63	0.84	294.50	1.00	5	Yes
Tramadol	22	55	0.89	324.00	0.67	4	Yes
Risperidone	8	37	0.97	816.00	0.36	4	Yes
Alprazolam	6	33	0.91	237.00	0.61	3	No
Clozapine	9	32	0.87	120.00	0.52	3	No
Lorazepam	6	30	0.81	0.00	0.84	3	No
Amiodarone	5	24	0.93	457.00	0.20	2	Yes
Codeine	13	22	0.83	108.00	0.26	5	No
Quetiapine	11	21	0.91	258.00	0.21	6	No
Zopiclone	3	21	0.85	114.00	0.36	2	No
Rilmenidine	5	20	0.96	500.00	0.49	4	Yes
Haloperidol	5	18	0.94	0.00	0.49	4	No
Acenocoumarol	5	15	0.79	379	0.02	1	No
Carbamazepine	8	15	0.75	142.00	0.07	4	No

^1^ Degree—the number of drugs a certain agent interacts with. ^2^ Weighted degree—the total number of interactions a drug presents in our analysis of X and D severity interactions. ^3^ Closeness—how close a drug is to all other drugs in the network of interactions. ^4^ Eigenvector—the higher the number, the more important drugs a certain drug is connected to. Describes drugs that act as hubs. Clinically, high numbers indicate drugs embedded in interconnected interaction clusters where theoretically multiple DDIs could occur. ^5^ Number of clusters connected—how many distinct communities a certain drug has in its proximal neighbourhood. The presence of these drugs raises the possibility that a drug causes a cascade of complex interactions because it connects numerous drug classes. ^6^ Bridge drug—these drugs are important drugs (high betweenness [≥90th percentile] and local neighbourhood connects at least two clusters). These are key drugs that are projected to be the most important in clinical practice.

## Data Availability

The data presented in this study are available on request from the corresponding author due to ethical and privacy restrictions.

## Supplementary Materials

The following supporting information can be downloaded at: https://www.mdpi.com/article/10.3390/medsci14030359/s1, Figure S1: Heatmap representation of the association of different secondary risk factors (disease states) and the major clinical outcomes (*n* = 76); Figure S2: Hierarchical clustering of the association of different risk factors and the major clinical outcomes (*n* = 76); Table S1: The basic characteristics of included and excluded patients; Table S2: Absolute number of patients suffering from the four clinical outcomes in the study; Table S3: Association analysis between exposure and outcome measures; Table S4: Association analysis between selected pairs of exposures and adverse clinical outcomes using the imputed dataset for exploratory secondary analysis (*n* = 275); Table S5: The results of the whole network of drug-drug interactions (i.e., all DDIs with no restriction on severity grades—A-X).

## Abbreviations

The following abbreviations are used in this manuscript:

NHR	Nursing Home Resident
DDI	Drug–Drug Interaction
HDI	Herb–Drug Interaction
PIM	Potentially Inappropriate Medication
OTC	Over The Counter
