# Supplementary material for: Network-Based, Cross-Sectional Analysis of Drug-Related Problems Reveals a Strong Association of Possible Inappropriate Medication and Clinical Outcomes in Romanian Elderly Nursing Home Residents
Source: Med Sci (Basel). 2026 Jun 29;14(3):359. doi: 10.3390/medsci14030359 (PMC13413638; doi:10.3390/medsci14030359)
Supplement: Supplementary file 1 [file medsci-14-00359-s001.zip › medsci-4340815-supplementary.pdf]

Supplementary materials: Network-Based, Cross-Sectional Analysis of Drug-Related Problems Reveals a Strong Association of Possible Inappropriate Medication and Clinical Outcomes in Romanian Elderly Nursing Home Residents

László-István Bába , Hanna Sebesi , Zsolt Gáll , Melinda Kolcsár, Soma Dávid , Noémi Medvés and George Jitca

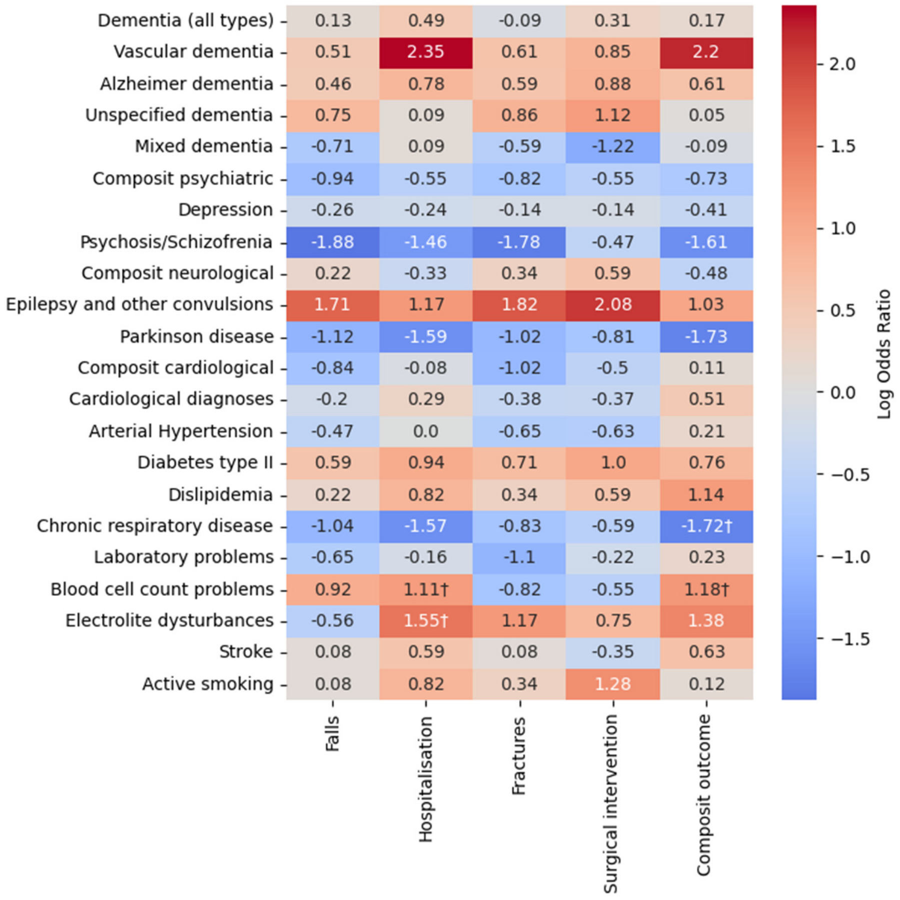

Supplementary Figure S1. Heatmap representation of the association of different secondary risk factors (disease states) and the major clinical outcomes (*n= 76*). Red - positive association (log OR >0 - OR>1); blue - negative association (log OR <0 - OR<1). Benjamini-Hochberg correction for false discovery rate has been applied. † associations close to significance (p between 0.06 and 0.09).

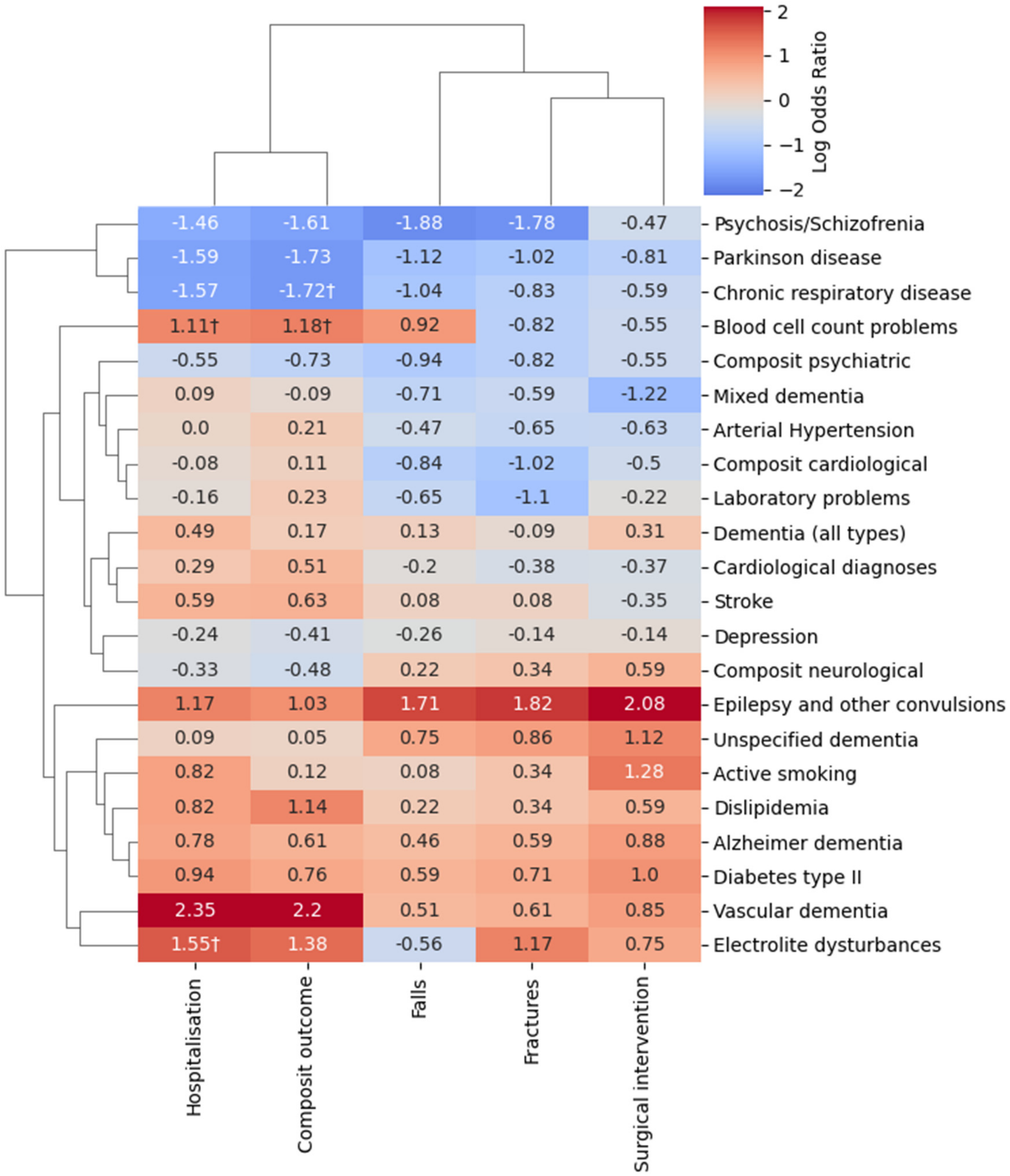

Supplementary Figure S2. Hierarchical clustering of the association of different risk factors and the major clinical outcomes (*n*= *76*). Agglomerative hierarchical clustering was used with euclidean distance metric and average linkage (UPGMA). Red - positive association (log OR >0 that is OR>1); blue - negative association (log OR <0 that is OR<1). Benjamini-Hochberg correction for false discovery rate has been applied, adjusted *p<0.05; ** p< 0.01; † p=0.09.

Supplementary Table S1. The basic characteristics of included and excluded patients.

	Included	Excluded	Statistical difference
Males	33	58	Chi squared test, X^2=5.062, df = 1, p = 0.0245
Females	43	141	
Age[1]	78.5 (69 - 84)	83 (75 - 88)	Mann-Whitney U = 5530, p=0.0005
Number of drugs[1]	8 (6-11)	10 (6-12)	Mann whitney U = 6540, p = 0.0822
Number of interactions per subject[1]	7.8 (3-13)	8 (3-15)	Mann Whitney U = 7106, p = 0.439
Morbidity (average number of diseases per NHR)[2]	3.42 ± 0.23	2.89 ± 0.13	Unpaired T test; T=2.139, p=0.03

[1]Medians with IQR

Supplementary Table S2. Absolute number of patients suffering from the four clinical outcomes in the study.

	No STOPP/START breach (n [% of patients without breaches])	At least one STOPP/START breach (n [%])
Falls	3 (6.12)	10 (37.04)
Fractures	3 (6.12)	9 (33.33)
Hospitalizations	9 (18.37)	10 (52.63)
Surgical intervention	4 (16)	6 (11.76)
Composite clinical outcome	12 (46.15)	9 (18.37)

Supplementary Table S3. Association analysis between exposure and outcome measures. Odds ratios, unadjusted and adjusted p-values (Benjamini-Hochberg method) are shown (n=76).

Exposure	Outcome	p	Adjusted p value	OR
STOPP/START PIM	Falls	0.001	0.009	9.02
STOPP/START PIM	Fractures	0.003	0.025	7.67
Blood cell count problems	Hospitalization	0.041	0.061	3.05
Electrolyte disturbance	Hospitalization	0.038	0.061	4.73
Blood cell count problems	Composite outcome	0.027	0.081	3.26
Chronic respiratory disease	Composite outcome	0.097	0.097	0.18

Supplementary Table S4. Association analysis between selected pairs of exposures and adverse clinical outcomes using the imputed dataset for exploratory secondary analysis (n=275). Odds ratios, unadjusted and adjusted p-values (Benjamini-Hochberg method) are shown.

**Exposure**	**Outcome**	**p**	**Adjusted p value**	**OR**
STOPP/START PIM	Falls	0.002	0.024	3.96
STOPP/START PIM	Fractures	0.096	0.315	2.21
STOPP-START PIM	Surgical intervention	<0.001	<0.001	47.70
STOPP-START PIM	Composite outcome	<0.001	<0.001	11.3
Blood cell count problems	Hospitalization	<0.001	<0.001	6.64
Electrolyte disturbance	Hospitalization	0.001	0.015	4.53
Blood cell count problems	Composite outcome	0.004	0.033	3.31
Chronic respiratory disease	Composite outcome	0.011	0.061	3.24
More than 8 drugs/supplements	Fall	0.0057	0.0476	2.163
Diabetes II	Fracture	0.0004	0.0064	2.996
Blood cell count problems	Hospitalization	<0.0001	0.0001	6.643

Supplementary Table S5. The results of the whole network of drug-drug interactions (i.e. all DDIs with no restriction on severity grades - A-X). The first fifteen drugs are shown in descending order of the weighted degree. Closeness centrality was omitted, since in the large graph there are numerous distinct unconnected clusters (the graph is disconnected), therefore the shortest paths become infinite.

Drug	Degree[1]	Weighted degree[2]	Betweenness[3]	Eigenvector[4]	Number of clusters connected[5]	Bridge-drug[6]
Risperidone	53	168	3,412,964,174.01	1.00	9	TRUE
Furosemide	60	167	31,764,060.28	0.70	9	FALSE
Alprazolam	42	144	878,961,173.40	0.71	7	TRUE
Quetiapine	47	138	861,569.37	0.85	9	FALSE
Indapamide	46	127	214,657,778.22	0.47	9	TRUE
Aspirin	37	111	3,964.67	0.35	9	FALSE
Levomepromazine	39	105	4,486.47	0.57	9	FALSE
Metoprolol	34	104	2,051.24	0.63	9	FALSE
Bisoprolol	35	103	1,730.59	0.56	9	FALSE
Tiapride	34	101	39,087,446.20	0.68	7	FALSE
Tramadol	44	99	69,382,696.70	0.48	9	TRUE
Clozapine	28	95	69,674,279.34	0.67	8	TRUE
Olanzapine	38	86	223,741,597.09	0.37	9	TRUE
Zopiclone	32	78	23,534.48	0.43	7	FALSE
